# Feasibility Study of an Optical Caustic Plasmonic Light Scattering Sensor for Human Serum Anti-Dengue Protein E Antibody Detection

**DOI:** 10.3390/diagnostics7030047

**Published:** 2017-08-17

**Authors:** Antonio A. García, Lina S. Franco, Miguel A. Pirez-Gomez, José L. Pech-Pacheco, Jorge F. Mendez-Galvan, Carlos Machain-Williams, Lourdes Talavera-Aguilar, José H. Espinosa-Carrillo, Miriam M. Duarte-Villaseñor, Christian Be-Ortiz, Luz E. Espinosa-de los Monteros, Ariel Castillo-Pacheco, Julian E. Garcia-Rejon

**Affiliations:** 1 School of Biological and Health Systems Engineering, Arizona State University, Tempe, AZ 85287, USA; tony.garcia@asu.edu; 2 School of Molecular Sciences, Arizona State University, Tempe, AZ 85287, USA; 3 Solexvintel S.A. de C.V., Ciudad de Mexico 01219, Mexico; Miguel@solexvintel.com.mx (M.A.P.-G.); joseluis@solexvintel.com.mx (J.L.P.-P.); jose.hector@solexvintel.com.mx (J.H.E.-C.); Programador3@solexvintel.com.mx (M.M.D.-V.); espinosaluzelena@hotmail.com (L.E.E.-d.l.M.); 4 Hospital Infantil de México Federico Gomez, Secretaria de Salud, Ciudad de Mexico 6720, Mexico; Jorge.f.mendez@gmail.com; 5 Laboratorio de Arbovirología, Centro de Investigaciones Regionales Dr. Hideyo Noguchi, Universidad Autonoma de Yucatán, Mérida 9709, Mexico; carlos.machain@correo.uady.mx (C.M.-W.); talaveralourdes@gmail.com (L.T.-A.); julian.garcia@correo.uady.mx (J.E.G.-R.); 6 Hemolab Scientific S.A. de C.V., Merida 97100, Mexico; beortizchristian@gmail.com (C.B.-O.); drjosearielcastillo@hotmail.com (A.C.-P.)

**Keywords:** Dengue, gold nanoparticles, optical caustic, protein E, light scattering

## Abstract

Antibody detection and accurate diagnosis of tropical diseases is essential to help prevent the spread of disease. However, most detection methods lack cost-effectiveness and field portability, which are essential features for achieving diagnosis in a timely manner. To address this, 3D-printed oblate spheroid sample chambers were fabricated to measure green light scattering of gold nanoparticles using an optical caustic focus to detect antibodies. Scattering signals of 20–200 nm gold nanoparticles using a green laser were compared to green light emitting diode (LED) light source signals and to Mie theory. The change in signal from 60 to 120 nm decreased in the order of Mie Theory > optical caustic scattering > 90° scattering. These results suggested that conjugating 60 nm gold nanoparticles and using an optical caustic system to detect plasmonic light scattering, would result in a sensitive test for detecting human antibodies in serum. Therefore, we studied the light scattering response of conjugated gold nanoparticles exposed to different concentrations of anti-protein E antibody, and a feasibility study of 10 human serum samples using dot blot and a handheld optical caustic-based sensor device. The overall agreement between detection methods suggests that the new sensor concept shows promise to detect gold nanoparticle aggregation in a homogeneous assay. Further testing and protocol optimization is needed to draw conclusions on the positive and negative predictive values for this new testing system.

## 1. Introduction

The development of a rapid and accurate means for identifying the cause of a systemic infection has been a subject of intense interest due to the need for proper course of treatment in order to improve patient outcomes. Additionally, there is a growing need for accurate diagnoses due to the toll in human and economic terms caused by endemic diseases transmitted by mosquitoes in tropical regions. Of particular focus in developing countries in the tropical region is the ability to ascertain whether a patient has contracted Dengue virus and specifically which serotype (DEN-1, 2, 3, 4) is the probable cause of the illness. While symptomatic diagnostics play an important role in all febrile disease management, some patients are asymptomatic or have mild symptoms, and diagnosis of these patients can be critical because Dengue Fever serotypes are believed to be directly linked to severe courses of infection such as Dengue Shock syndrome (DSS) and Dengue Hemorrhagic Fever (DHF), making early serotype identification a potentially valuable tool. Moreover, confirmation of whether the patient has had a prior serotype infection, which has also been linked to DSS and DHF, can also help to determine the course of treatment and further steps to prevent an outbreak of Dengue [1,2,3,4].

In order to meet the need for a rapid diagnostic that can be implemented in the field and closely based on patient exposure and/or immune response at an early stage of patient-reported symptoms, a hybrid approach that combines gold nanoparticle conjugates (similar to what is used in low-cost paper diagnostics based on lateral flow immunoassay technology) in conjunction with a portable sensor for detecting a positive response with a high level of sensitivity at low antigen levels is the subject of this feasibility study. In order to establish a relatively low-cost diagnostic tool, the hybrid technology described in this paper builds upon the employment of an optical caustic light scattering technology that employs no lenses and does not need filters [5]. Moreover, the use of battery-powered LEDs and the deployment of a smartphone to analyze the scattered light are also helpful in reducing costs, as well as in improving access to the device for low and middle income countries.

To commence a process of verifying the capabilities of the new hybrid technology platform in a simulated low resource setting, our approach was to first determine if an existing Dengue molecular reagent used for gold nanoparticle conjugation in paper assays could detect Dengue antibodies with human patient samples by employing this new hybrid technology platform. For the first part of this study, we analyzed the 90° light scattering response of conjugated gold nanoparticles to different concentrations of anti-protein E antibody in vitro to determine key parameters, such as: (1) the size of the gold nanoparticle that should be used; and (2) the stability of the gold nanoparticle conjugates when subjected to transportation and varying temperature conditions. For the second part of the study, a direct approach with clinically-relevant human serum samples, was used since it is a faster way to determine other important parameters such as: (1) whether very small amounts of human serum samples can yield reasonable signals; (2) determine the need for additional dilution or blocking steps when working with human serum; and (3) whether room or body temperature and incubation time generate differences in measurement. Thus, the overall objective of this feasibility study is solely to gain experience in practical aspects of developing a rapid and hybrid quantitative assay for determining Dengue infection by monitoring patient antibody response while simulating a low resource setting in order to expand testing in a variety of locales and with a wider variation in infrastructure than is normally the case in high-income country clinics and hospitals.

## 2. Materials and Methods

### 2.1. Gold Nanoparticle Light-Scattering Calibration

For calibration purposes, unconjugated gold nanoparticles of 20, 40, 50, 60, 80, 100, and 200 nm (Ted Pella Inc., Redding, CA, USA) were diluted 1:10 in distilled deionized water and analyzed for light scattering and extinction using three separate detection methods. Each gold particle size sample contained the same overall molar concentration of gold. Light extinction spectra acquisition were taken with an Ocean Optics Fiber Optic Spectrometer (Ocean Optics, Dunedin, FL, USA, http://oceanoptics.com) specifically a USB4000 spectrometer and SpectraSuite software (Ocean Optics, Dunedin, FL, USA, http://oceanoptics.com) for control and data acquisition. A quartz cuvette of 1 cm path length was used to acquire samples. Spectra for ultraviolet-visible (UV-VIS) data was generally collected between 10 and 100 ms integration time and 20 and 100 spectra were averaged for each sample.

### 2.2. Gold Nanoparticle Conjugation

In a 1.5 mL Eppendorf tube, an aliquot of 1 mL containing 2.6 × 10^10^ spherical particles/mL (37.7 pM) of 60 nm citrate stabilized gold colloids (Ted Pella Inc., Redding, CA, USA) was combined with 100 microliters of freshly prepared 1 M pH = 8.5 borate buffer, which has the same pH as the protein E, prior to the addition of protein E Dengue Envelope-2 32 kDA (MCR-054, Reagent Proteins, Pfenex Inc., San Diego, CA, USA). The amount of protein E was established performing a titration with different amounts of protein E and salt addition to determine the amount of protein needed when colloid stability is reached, as it was described before [6,7]. Aggregation of the gold nanoparticles was observed when the amount of protein was not enough, and it was evidenced by visual aggregation and color change from pink to purple or a clear solution. Three different amounts of protein E were conjugated to 1 mL of 60 nm gold nanoparticles, showing that at least 42.5 µg were needed to cover the gold nanoparticles and not induce aggregation in the presence of PBS after the last re-suspension. Fifty microliters of a 0.85 mg/mL Dengue Protein E solution were added to the solution of borate buffer and gold nanoparticles described above. A frozen cold pack was placed on the tube and the mixture was gently rocked using an orbital table for 30 min. This was followed by the addition of 2.5 microliters of 10% Tween 20 and rocking on the orbital table for five more minutes.

One or three centrifugation steps were then conducted in order to reduce the concentration of unbound protein E form the suspension. A Beckman Coulter Microfuge 18 (Beckman Coulter, Brea, CA, USA) was placed in a 4 °C environmental room in order to minimize aggregation of gold nanoparticles and ensure resuspension after centrifugation. The first centrifugation was for 30 min at 3500× *g*. Immediately after centrifugation, as much of the supernatant as possible was carefully removed from the pellet using a 200 µL micropipette, while not disturbing the pellet. Once this was accomplished, 1 mL of 1 M pH = 8.5 borate buffer was added to re-suspend the pellet. An aliquot of 2.5 microliters of 10% Tween 20 was then added to the suspension and the mixture was gently rocked for 5 min. Afterwards, the suspension was centrifuged again for 15 min at 3500× *g*. The same procedure was used following the second centrifugation as described for the first centrifugation step. For the third centrifugation, the time was shortened to 10 min. After the third and final centrifugation, the pellet was re-suspended in 0.5 mL of a 0.2 mg/mL solution of bovine serum albumin (BSA) in deionized (DI) water to block the remaining unreacted sites on the gold nanoparticles. The particles were then incubated on the orbital table for an additional 5 min. This was followed by the addition of 0.5 mL of DI water and 0.5% sodium azide. The final concentration of the conjugated gold nanoparticles was calculated using the absorbance measured using a NanoDrop 2000 (Bio Rad Laboratory, Hercules, CA, USA) and the molar extinction coefficient, and it was 1.19 × 10^10^ particles/mL. The stability and confirmation of the conjugation of the gold nanoparticles was monitored using UV-VIS spectrophotometry using a Nanodrop 1000 (Bio Rad Laboratory, Hercules, CA, USA) and dynamic light scattering measurements.

### 2.3. Detection of Anti-Protein E Antibody In Vitro

To 100 µL of the conjugated gold nanoparticle solution (1.19 × 10^10^ particles/mL), 900 µL of phosphate buffer saline (PBS) were added. Posteriorly, 10 µL of different concentrations of anti-protein E antibody (0–10 ng/mL) were added prior to measuring 90-degree light scattering using the oval plastic chamber and the handheld optical caustic sensor which used a Nokia 920 smartphone and Lumia software for imaging. Pictures of the oval chamber were taking using different times of exposure (0.1 s or 1 s) and by reducing overall brightness to the lowest level in the Lumia camera app. The images were analyzed measuring the raw intensity in ImageJ (NIH, Bethesda, MD, USA) using the green or red channel, depending upon the level of saturation of the green channel, and by focusing in the zone of the sample chamber where scattering by the gold nanoparticles provides pixels with brightness above the typical PBS solution pixel values of 0–5 (out of a maximum range of 255). The controls were compared with the samples for statistical differences using the two-tailed *t*-test.

### 2.4. Dot Blot Using Conjugated Gold Nanoparticles

Standard dot blot paper (Bio Rad Laboratories, Hercules, CA, USA) was cut into rectangular or hexagonal shapes in order to fit into 12 well Corning Costar Tissue Culture Plates (Ted Pella Inc., Reading, CA, USA). After punching a hole in the center of the paper with a sterile pin, 3 µL of 1:100 PBS diluted human serum was placed over the area around the pin hole and allowed to dry for 5 min at 37 °C. Then, the paper was cooled to 4 °C and allowed to incubate for 15 min. Following incubation, an aliquot of 1 mL of a 3% skim milk in PBS blocking solution was added to the chamber and mixed for 15 min at room temperature. After incubating in the blocking solution for 30 min at 37 °C, an aliquot of 500 µL of a 1:10 diluted suspension of 60 nm protein E conjugated gold nanoparticle was added to the paper. Incubation with the gold nanoparticle conjugate was conducted for 1 h at 37 °C. After the final incubation, the paper was rinsed three times in PBS–Tween 20 buffer and allowed to dry.

### 2.5. Testing of Human Serum Samples

The Committee of Bioethics from the Regional Research Center, and Dr. Hideyo Noguchi at the Autonomous University of Yucatan (UADY), approved the use of sera samples previously obtained for another research project (CIRB-2010-0021, 1 December 2010). The samples were obtained between 2010 and 2015 by medical personnel. The frozen stored samples coded and linked to their test results were donated in a depersonalized manner, making sure no researcher was involved in the identification process of any sample.

Two 6 mL bottles of conjugated gold nanoparticle reagent gold nanoparticles (GNP)-60-E were created (1.19 × 10^10^ particles/mL) according to the protocol described above except that while one GNP reagent (GNP-60-E-3) was centrifuged three times to remove unbound protein E, a second reagent (GNP-60-E-1) was centrifuged only once. Both reagents were stored for 16 h at 4 °C then transported at room temperature for 19 h, until refrigerated at 4 °C at the UADY. Both GNP-60-E reagents arrived un-aggregated at UADY based on both Nanodrop 2000 spectrometer (Bio Rad Laboratory, Hercules, CA, USA) visible spectra taken at the Universidad Autónoma del Yucatán before using the reagents and by visual observation of the gold nanoparticle suspension.

For each test, in a 1.5 mL Eppendorf tube, 900 µL of PBS were combined with 100 µL of the gold nanoparticle conjugate solution followed by the addition of 5 or 10 µL of the human serum samples or a negative control of PBS. For the testing at 37 °C, GNP-60-E-3 was used and 10 microliters of undiluted human serum was added. Incubation was performed for 30 min at 37 °C without agitation. Afterwards, each test sample was placed in an oblate spheroid chamber and capped, followed by imaging with the smartphone sensor. For the first trial at 22 °C, GNP-60-E-3 was used and 5 µL of undiluted human serum was added. Incubation was performed for 15 min at 22 °C without agitation. Afterwards, each test sample was placed in an oblate spheroid chamber and capped, followed by imaging. For the second trial at 22 °C, GNP-60-E-1 was used and 2 µL of 1:10 diluted human serum was added. Incubation was performed for 15 min at 22 °C without agitation. Afterwards, each test sample was placed in an oblate spheroid chamber and capped, followed by imaging. Light scattering was analyzed using ImageJ using the average gray values obtained in vitro with the controls as a reference for positive and negative values. Negative values would be classified as the negatively value plus two standard deviations, positive if it was close to the positive control, and weakly positive if it was a mean gray value in the middle.

### 2.6. Light Scattering Measurement Systems

For 90-degree laser light benchtop scattering measurements, an Ocean Optics Fiber Optic Spectrometer (Ocean Optics, Dunedin, FL, USA) was used with a USB4000 spectrometer and SpectraSuite Software for control and data acquisition. A quartz cuvette of 1 cm path length with all four windows clear for measurement enables 90-degree placement of an InPhotonics 532 nm laser, with an Ocean Optics controller from the fiber optic collector, to measure counts of scattered light. Readings of 1 s integration time with five spectra averaging was used.

For dynamic light-scattering measurements, a Beckman Coulter DelsaNano C particle analyzer (Fullerton, CA, USA) was used to perform size analysis (with 50 measurements over 2 s for each sample) with CONTIN, Marquandt or non-negative least squares (NNLS) analysis to obtain the intensity distribution.

### 2.7. 3D Printed Oblate Spheroid Chamber

Autodesk Fusion 360 software was used to generate the 3D oblate spheroid sample chamber used in the handheld optical caustic sensor. In order to place the sample chamber in the LED illumination chamber and easily remove it after imaging, a solid rectangular peg was added to the center of the base. The opening of the sample chamber was sized and designed with a small ridge in order to accommodate lids cut from 200 microliter PCR tubes. Fabrication of the sample chambers were done in batches of 12 by transmitting the drawing to a 3D printing company (iMaterialise, Leuven, Belgium). 3D printing is conducted using stereolithography with a transparent resin and the finished product is between water clear and translucent, in terms of visibility to the naked eye.

### 2.8. Handheld Smartphone Optical Caustic Light Scattering Sensor

A previously described handheld smartphone-enabled optical caustic light scattering sensor was used to capture images of samples in an oblate spheroid chamber [5,8]. A 532 nm green photodiode (Industrial Fiber Optics, Tempe, AZ, USA) illuminated the sample chamber at a 90° angle from a Nokia Lumia 920 (Nokia, Espoo, Finland) smartphone camera lens. Images were collected at 0.5 s exposure using the Lumia camera app. At this exposure level, and with the intensity of light used, the green channel of the Red, Green, Blue color model (RGB) images saturate. However, excess light due to scattering can be imaged in the center of the sample chamber by splitting the color channels using ImageJ. ImageJ software was used to collect the intensity of the red, green, and blue channels for each image. Integrated density measurements from ImageJ was collected for a constant image area and used to quantify the amount of scattering observed for gold nanoparticles in water, conjugated gold nanoparticles with a serum sample, or a phosphate-buffered saline (PBS) control.

### 2.9. Digital Color Optical Caustic Light-Scattering Sensor

In lieu of a smartphone to collect images and in order to corroborate the gold nanoparticle scattering vs. size calibration with the optical caustic sample chamber, a digital color sensor S9706 (Hamamatsu Photonics, Hamamatsu, Japan) collected light from the oblate spheroid sample chamber at a 90° angle and a distance of 10 mm from the center of the chamber. Due to the proximity of the sensor to the sample chamber, a 3D printed tubular mask with an opening of 7 mm in diameter was used to block the incident light. A 3D printed chamber with interior angled walls was also fabricated and used to minimize light reflection. The digital color sensor was connected to an Arduino Uno computer and the manufacturer’s recommended algorithm was deployed to acquire data at different integration times. Data for the gold nanoparticles in water was acquired using between 0.1 and 30 s integration time. Since a lower intensity of LED light was used than with the smartphone system, data for 20, 40, 50, 60, 80, 100, and 200 nm was collected for the green channel of the sensor. The most accurate data to compare scattering using the digital color sensor in this system was the highest integration time of 30 s, which was then used to compare with the smartphone light-scattering results.

## 3. Results

### 3.1. Gold Nanoparticle Size Suitable for the Assay

Before conjugation of protein E to gold nanoparticles, the choice of gold nanoparticle size was deemed to be a key variable that needed to be determined. Gold nanoparticles are known to have optical properties such as light extinction and light scattering that change with the size of the particle. To characterize the change in surface plasmon as size increases, light extinction spectra of 20, 40, 50, 60, 80, 100, and 200 nm gold nanoparticles were measured. The plasmon peak of the gold nanoparticles moves to the infrared wavelength, from 520 to 580 nm, as the size of the gold nanoparticle increases (Figure S1). The homogeneity of the gold nanoparticles in solution was confirmed by measuring the polydispersity index (PDI = 0.077 < 0.1) and the hydrodynamic radius confirmed the sizes of the gold nanoparticles.

**Figure 1:**
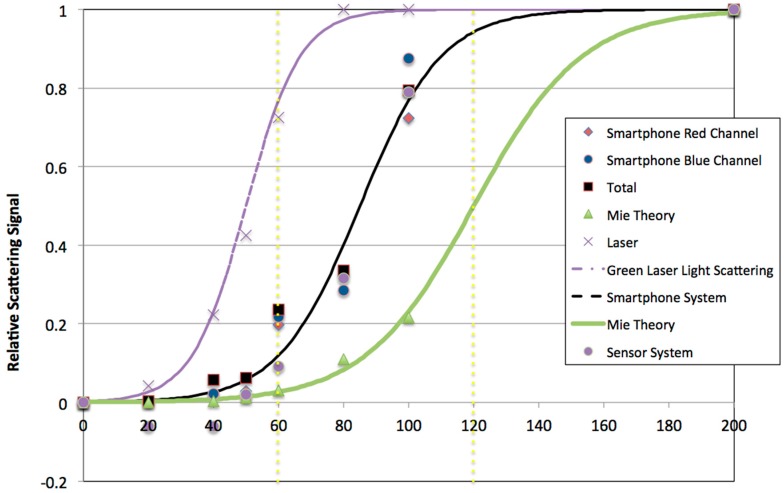
Comparison of gold nanoparticle scattering with green light data for a laser and fiber optic spectrometer, LED/optical caustic smartphone, and LED/optical caustic digital color sensor. Discrete calculations of the total scattering at 532 nm using Mie theory is also shown. Data and calculations are normalized to the maximum value for gold nanoparticles of 200 nm and fitted to a logistic curve. Horizontal dashed lines illustrate the signal difference for 60 and 120 nm diameter gold nanoparticles.

Based on the well understood properties of gold nanoparticles to light scattering via plasmon resonance [9] near the maximum for spheres (520–540 nm), a calibration was conducted with a laser light scattering fiber optic spectrometer, the optical caustic sensor, and calculations based on Mie Theory [9,10]. Figure 1 illustrates two key points. First, that the laser light scattering system which is based on 90° scattering is sensitive to small changes in particles size at a narrow range of between 30–70 nm diameter particles. Mie Theory calculations illustrate that the total scattering for gold nanoparticles increases more gradually with size, since this calculation takes into account all angles of scattering. The optical caustic sample chamber is seen in Figure 1 to fall between the two groups of data, using both a camera and a digital color sensor to collect data. It was also found that the red channel of the smartphone RGB image was most useful based on the LED intensity and shutter speed setting since smartphone sensor saturation “bleeds” signal to the red and blue channels when there is an excess of green in the image. The digital color sensor corroborates that interpretation, as shown in Figure 1.

### 3.2. Detection of Anti-Protein E Antibody In Vitro

After establishing that 60 nm gold particles would be a useful size, it is also important to note that conjugated gold nanoparticles are sensitive optically to any surface change, including binding of antibodies or antigens to their surfaces and the additional binding of proteins to their surfaces or aggregation of gold nanoparticles. The conjugated gold nanoparticles were characterized by measuring the light extinction spectra and the hydrodynamic radius of the samples (Figure S2). These confirmed an increase in the hydrodynamic radius of the particle suggesting that the protein E was conjugated, and was confirmed by the light extinction spectrum of the conjugated gold nanoparticles, where the plasmon peak shifted to the infrared wavelength (536 to 544 nm).

**Figure 2:**
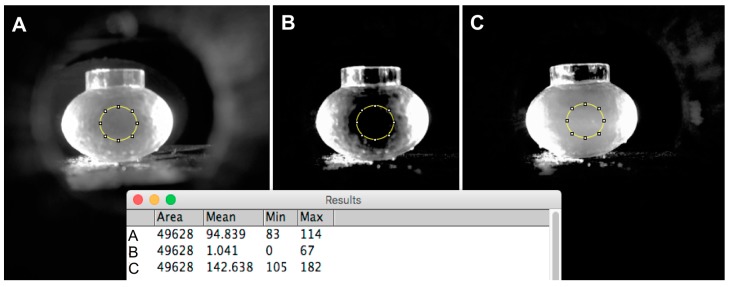
Light scattered using PBS or gold nanoparticles in the oval chamber. (**A**) Light scattering intensity of PBS measured with the green channel of a picture taken with 0.1 s of exposure time; (**B**) Light-scattering intensity of PBS measured with the red channel of a picture taken with 1 s of exposure time; (**C**) Light scattering intensity of 60 nm conjugated gold nanoparticles measured with the red channel of a picture taken with 1 s of exposure time. The table in the figure represent the gray values for the area inside the circle for each sample.

To establish the response of the system in terms of 90° light scattering near the plasmon resonant peak, different amounts of anti-protein E antibody were added to a diluted solution of protein E conjugated gold nanoparticles. The antigen protein E used as the reagent conjugated to the gold nanoparticles is a recombinant protein with selectivity towards Dengue Serotype 2 antibodies. The first parameter to determine was the zone for no scattering in the chamber, and in this case PBS only without gold nanoparticles was used. Images taken with 1 s of exposure and analyzed using the red channel since the green channel was already saturated with green light (Figure 2A,B) shows that when using only PBS, the amount of light detected from the center of the sample chamber is very low since no scattering nor reflection is expected in this zone of the sample chamber. Other images using different exposure times with the green channel did not provide a sufficiently good baseline dark zone.

Next, images were taken for an aqueous suspension of 60 nm gold nanoparticles using the same imaging settings as for PBS. Figure 2C shows that the region given as a dark area when imaging PBS now contains brightness due to gold nanoparticle light scattering.

Finally, in order to quickly explore the range of signal change possible for a commercially available protein E antigen with the handheld optical caustic sensor, the light-scattering intensity for a gold nanoparticle suspension and gold nanoparticles containing protein E in the presence of 10 µg/mL of anti-protein E antibody in PBS. Table 1 gives the integrated density differences for the controls and the antibody test. The mean gray value for the conjugated gold nanoparticles in the presence of the anti-protein E antibody shows more light scattered due to the aggregation caused by the presence of the antibody with the gold nanoparticles.

### 3.3. Detection of Anti-Protein E Antibody in Human Serum Samples

The initial challenge lay in interpreting patient results without an extensive study of the antigen protein structure nor its immunogenic properties when presented on gold nanoparticle surfaces. This makes discerning the performance of the tests with respect to patient Dengue status characterization challenging. The challenge is somewhat reduced by using a dot blot test with the same patient samples and gold conjugate reagents as an orthogonal test. Table 2 provides information on the 10 patient serum samples donated by the Arbovirus laboratory of UADY for this study. These samples represent a spectrum of patients including Dengue negative, Dengue Serotype 2 positive with a first-time (primary) infection and second-time (secondary) infection, patients convalescing from a Dengue Serotype 2 infection, and patients with Dengue Serotypes 1 and 3. The antigen protein E used as the reagent conjugated to the gold nanoparticles is described by the manufacturer as being most sensitive to immunoglobulin M (IgM) levels, although some cross-reactivity can present with other serotypes [1,2,3,4].

**Figure 3:**
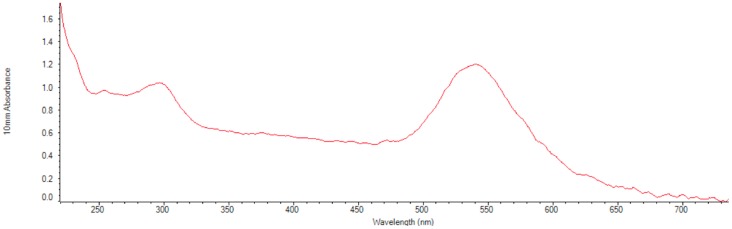
Nanodrop spectrometer UV-VIS spectra. The absorbance in the UV is due to the BSA blocking solution. The absorbance maxima at a wavelength of 540 nm is consistent with the expected plasmon resonant peak for conjugated gold nanoparticles of 60 nm diameter.

Before commencing with the patient samples testing, the 60 nm gold conjugates were inspected and the UV-VIS spectra were recorded for the two batches of particle conjugates (Figure 3). Both visual and spectral information verified that the gold conjugates were stable after all the changes in temperature (meaning the plasmon peak was stil 544 nm), and ready to use at UADY.

Once the stability of the gold nanoparticles was confirmed, detection of the anti-protein E antibody was performed by a modified dot blot protocol using gold nanoaparticles. Table 3 summarizes the expected dot blot score based on the protein E gold conjugate reagent (GNP-60-E-3) used, assuming some level of cross-reactivity with other Dengue serotypes and a 50% probability of measuring antibodies reactive to protein E after 120 days of convalescence compared to the individual blot samples with their score (Figure S3). It is observed that most of the samples agree between what was expected and what is the score of the dot blot. However, for the samples of Patients 1 and 2, the dot blot results gave a false positive.

**Figure 4:**
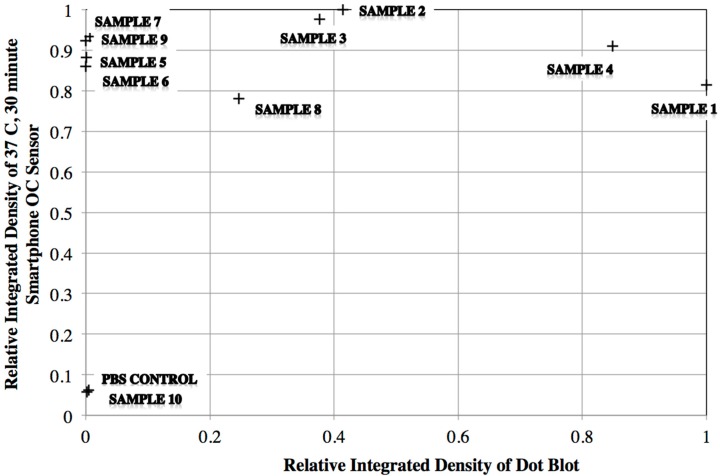
Comparison of dot blot and optical caustic smartphone sensor. Data were recorded at the same sample incubation temperature of 37 °C.

After analyzing the samples using the dot blot method, the samples were evaluated using the gold nanoparticles and the optical caustic smartphone sensor. In this case, different preparations of conjugated gold nanoparticles were tested to establish which preparation method (either three or one centrifugation step(s)) would generate the more stable gold nanoparticles, and different incubation times and temperatures with the samples. A summary of the three sets of tests with human serum samples and an overall score is given in Table 4 while a quantitative comparison of the dot blot is shown in Figure 4. Table 4 shows that the preparation method that generates the most accurate results is when the incubation with the samples occurs for 15 min at 22 °C with only one centrifugation step.

In Figure 4, there are two overall features to note. First, it appears that the smartphone signal saturates at low “signals” from the dot blot experiments. The second feature is that the negative control and patient sample 10 are both low for the dot blot and the optical caustic sensor, but there is disagreement between patient samples 5 and 6 between the two detection methods. Another observation is that patient sample 7 yielded a very weak signal, and patient sample 10 is negative based on the dot blot tests, yet the optical caustic sensor data show strong signals.

## 4. Discussion

Detection methods in developing countries for tropical diseases need to be accurate and performed in a timely manner to prevent disease. The approach described in this study aimed to combine gold nanoparticle conjugates with a portable (smartphone) sensor for detecting with a high level of sensitivity at low antigen levels [8]. The first part of the study described was the calibration of the gold nanoparticle size (Figure 1). The overall interpretation of the calibration data is that using 60 nm gold particles is very practical for the intended application. The reason for this is that there is a dramatic (approximately 340%) increase in signal predicted when two 60 nm gold particles aggregate upon antibody-antigen binding, basically the change in relative signal from 60 to 120 nm gold particle size.

The stability of the conjugated gold nanoparticles is a factor that directly affects the efficiency of the assay. The process of conjugation was first performed with one or three centrifugation steps in order to eliminate free protein that was not bound to the gold nanoparticles adding before each step a surfactant (Tween 20) to help resuspend the gold nanoparticles [10]. After the gold nanoparticles were conjugated with protein E, light extinction and DLS measurements were performed confirming the conjugation (Figure S2). The conjugated nanoparticles were then exposed to different temperatures during long periods of time (~36 h) and after characterized again, they showed they were stable by observing the plasmon peak at 544 nm in the light extinction spectrum. 

The second part of the calibration was to establish the response of the system in terms of 90° light scattering near the plasmon resonant peak. Images of PBS in the oblate chamber were taken using different exposure times and analyzed with the green channel, which did not provide a sufficiently good baseline dark zone. Images analyzed with the red channel provided a good baseline dark zone with PBS (Figure 2B), so the most appropriate exposure time for imaging for the handheld optical caustic smartphone was determined to be a 1 s exposure in the center zone of the images’ red channel. This was confirmed when the results obtained with PBS were compared to the image obtained with conjugated gold nanoparticles (Figure 2C). The integrated difference for these two measurements is on the order of 100×, which clearly provides a high range for detecting small changes in gold nanoparticle scattering. It is important to note that this signal is based on only 3 billion gold nanoparticles in 0.7 mL of suspension, which suggests that detection to a sensitivity of 50 pg/mL of antibody or antigen should be possible since it could represent a 1% difference in nanoparticle scattering signal. These conditions were then used when 10 µg/mL of anti-protein E antibody was added to the conjugated gold nanoparticles and a picture was taken which confirmed the sensitivity of the conditions used for the assay.

When working with human serum samples other components that are present in serum need to be taken into account when analyzing the sensitivity and specificity of the assay. In the case of Dengue, there are other factors that could affect sensitivity and specificity: if the sample is taken at an early stage or late stage of the disease, and the possible cross-reactivity with other antibodies Patients with early stage and primary dengue infection should present IgM against Dengue antigens after a few days of infection and the IgM levels should decrease after 90 days [1,2,3,4,11,12,13]. Patients with a secondary infection should mostly present IgG antibodies [1,2,3,4,13]. The antigen protein E used was from Dengue Serotype 2 and is described by the manufacturer as being most sensitive to IgM levels. However, it is known that there can be cross-reactivity to other Dengue serotype antibodies, as well as from patients who have experienced other arbovirus infections from the group known as flaviviruses, such as West Nile, St. Louis Encephalitis, Zika Virus, and Chikungunya [12,14]. These considerations suggest that there should be some variation in test results, but the variations may be difficult to discern without a more comprehensive series of human serum controls, which would be performed in the future.

The overall agreement between the dot blot score and the expected results is very good. However, the two Dengue-negative patients appear to give positive dot blot results. It is not clear why this is the case, but perhaps a current or prior flavivirus infection is being detected by the gold conjugate reagent. Additionally, the sample with PBS added and no human serum was correctly identified as a negative control in the dot blot experiment. It is also important to note that a dot blot test with a monoclonal antibody that reacts with flavivirus group specific antigens (4G2, KPL; Abcam, San Francisco, CA, USA) was found to be negative (not shown). The negative result suggests that epitopes of the protein envelope needed for the monoclonal antibody to bind to the GNP-60-E-3 were blocked due to the immobilization of protein E to the gold nanoparticles. This is a potentially useful observation for further gold conjugate development, namely if a methodology for protein E binding to gold based on covalent attachment rather than his-tag is used. For this study, it appears that the results with GNP-60-E-1 at 22 °C for 15 min seem to be most closely related to the characterization based on the dengue patient status information (Table 4).

When comparing the dot blot results with the smartphone optical caustic sensor results, as observed in Figure 4 there are two main features to note. The smartphone signal saturates at low signals compared to the dot blot, which seems reasonable given the high signal change described in Figure 1 and Figure 2 for aggregates consisting of 60 nm gold particles. The second feature is the differences in results of samples 5 and 6 for both methods. This may be due to the higher sensitivity of the optical caustic sensor as compared to the dot blot which used gold nanoparticles at a higher level of dilution. Dot blots are also considered to be lower in sensitivity due to diffusion and orientation effects when binding to a fixed surface. Additionally, it is important to mention the weak signals observed with dot blot of samples 7 and 10, which may be due to higher sensitivity of the optical caustic system and cross reactivity of the Dengue Serotype 2 recombinant protein E with other Dengue serotypes. As noted in the results for GNP-60-E-1 at 22 °C and 15 min seen in Table 4, it appears that this cross-reactivity may be diminished by using a higher dilution of human serum, lower temperature, and allowing some unbound antigen in the gold nanoparticle conjugation suspension.

## 5. Conclusions

A feasibility study of a hybrid gold nanoparticle with an optical caustic sensor system indicates that the technology is sensitive to the detection of serum antibodies against dengue fever. The use of similar size gold nanoparticles (60 nm) to what is often used in paper lateral flow rapid tests is justified based on calibration data and human patient sample testing. The gold nanoparticle conjugates are stable to transportation and temperature fluctuations and appear to be un-aggregated and active after several days of conjugation. Additionally, all negative controls for the dot blot and optical caustic sensors were correctly measured as negative.

Dot blot tests were found to be useful to verify the reactivity of the gold nanoparticle conjugates to patient serum antibodies and may also help identify epitope presentation differences when applying monoclonal antibodies to the dot blot paper directly. It appears that human serum should be diluted and that testing can be done at around 22 °C within 15 min using the hybrid gold nanoparticle conjugate reagent and optical caustic sensor. A more thorough investigation of well-characterized human serum samples is still needed in order to optimize the test conditions and buffer components and generate positive predictive and negative predictive values that reflect the capabilities of the diagnostic platform.

## Figures and Tables

**Table 1:** Average gray values as a light scattering measurement for samples. Mean gray values of samples of PBS, gold nanoparticles, diluted gold nanoparticles with PBS and 10 μg/mL of anti-protein E antibody, in the oval chamber. The average value is from 3–6 replicates.

Sample	PBS	Conjugated Gold Nanoparticles	Conjugated Gold Nanoparticles with PBS	Gold Nanoparticles with 10 µg/mL of Anti-Protein E Antibody
Area (a.u.)	49,628	49,628	49,628	49,628
Mean gray value (a.u.) ± S.D.	1.181 ± 0.737	146.214 ± 10.233	119.060 ± 12.750	159.800 ± 7.658 *

* Statistically significant (*p =* 0.0003) with respect to the conjugated gold nanoparticles with PBS.

**Table 2:** Description of human serum samples. Human serum samples used in the feasibility study with descriptive information on Dengue status.

Patient	Dengue Status	Description of Sample	Primary or Secondary Infection	Stage of the Disease
1	Negative	Asymptomatic, healthy individual, PCR-IgM-IgG negative for Dengue Virus	N.A.	N.A.
2	Negative	Asymptomatic, healthy individual, PCR-IgM-IgG negative for Dengue Virus	N.A.	N.A.
3 *	DENV-2	PCR positive for DENV-2, IgM positive for DENV-2	Primary	Initial stage
4 *	DENV-2	PCR positive for DENV-2, IgM positive for DENV-2	Primary	Initial stage
5	DENV-2	Seropositive for DENV-2, IgM negative for DENV-2, day 4	Secondary	Initial stage
6	DENV-2	Seropositive for DENV-2, IgM negative for DENV-2, day 4	Secondary	Initial stage
7	DENV-3	PCR positive for DENV-3	Unknown	Initial stage
8	DENV-1	PCR positive for DENV-1	Unknown	Initial stage
9 **	DENV-2	Volunteer donated sample, seropositive for DENV-2, IgM positive for DENV-2	Unknown	Convalescing > 120 days
10 **	DENV-2	Volunteer donated sample, seropositive for DENV-2, IgM positive for DENV-2	Unknown	Convalescing > 120 days

* Patients positive for Dengue with negative IgG test. ** Patients volunteered for testing were positive with DENV-2 during the epidemic in 2015 and donated samples four months after initial diagnoses. N.A.: Not Applicable; PCR: Polymerase chain reaction; IgM: Immunoglobulin M.

**Table 3:** Comparison of results of the human serum samples between diagnostic methods. Comparison of dot blot with expected result due to Dengue status reported by the clinical laboratory. The protein E used, while specific for DENV-2, has sensitivity for IgM detection and can have cross-reactivity with other serotypes.

Patient	Expected Dengue Result Based on Dengue Serotype and Some Cross-Reactivity (Assuming High Sensitivity to IgM)	Dot Blot Score ( + or −)	Agreement
1	−	+	No
2	−	+	No
3	+	+	Yes
4	+	+	Yes
5	−	−	Yes
6	−	−	Yes
7	−	−	Yes
8	+	+	Yes
9	+	+	Yes
10	−	−	Yes

**Table 4:** Qualitative summary of optical caustic scattering smartphone sensor data.

Patient	GNP-60-E-3 37 °C for 30 min	GNP-60-E-3 22 °C for 15 min Trial 1	GNP-60-E-1 22 °C for 15 min Trial 2	Overall Score (+ or −)
1	Weak positive	positive	negative	−
2	positive	negative	positive	+
3	positive	positive	positive	+
4	positive	positive	weak positive	+
5	positive	positive	positive	+
6	positive	negative	positive	+
7	negative	weak positive	negative	−
8	positive	positive	negative	−
9	positive	weak positive	positive	+
10	very weak positive	positive	positive	+
PBS control	negative	negative	negative	Negative control
PBS control	negative	negative	negative	Negative control

GNP: gold nanoparticles.

## Supplementary Materials

The following are available online at www.mdpi.com/2075-4418/07/3/47/s1, Figure S1. Light extinction spectra of different sizes of gold nanoparticles; Figure S2. Characterization of the conjugated gold nanoparticles; Figure S3. Image showing visual scoring of dot blots.
