# Supplementary material for: Feasibility Study of an Optical Caustic Plasmonic Light Scattering Sensor for Human Serum Anti-Dengue Protein E Antibody Detection
Source: Diagnostics (Basel). 2017 Aug 17;7(3):47. doi: 10.3390/diagnostics7030047 (PMC5617947; doi:10.3390/diagnostics7030047)
Supplement: Supplementary file 1 [file diagnostics-07-00047-s001.zip › diagnostics-212526-supplementary/Suppl Figures/Figure S1.pdf]

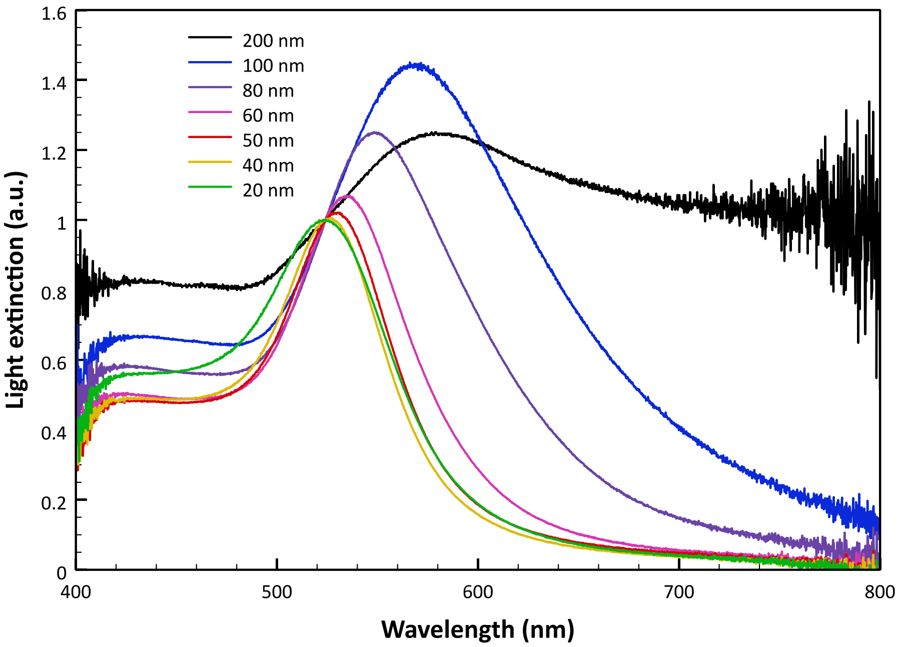

Figure S1. Light extinction spectra of different sizes of gold nanoparticles. As the size of the gold nanoparticle increases, the plasmon peak shifts to the infrared wavelength and the light extinction increases.
