# Supplementary material for: Feasibility Study of an Optical Caustic Plasmonic Light Scattering Sensor for Human Serum Anti-Dengue Protein E Antibody Detection
Source: Diagnostics (Basel). 2017 Aug 17;7(3):47. doi: 10.3390/diagnostics7030047 (PMC5617947; doi:10.3390/diagnostics7030047)
Supplement: Supplementary file 1 [file diagnostics-07-00047-s001.zip › diagnostics-212526-supplementary/Suppl Figures/Figure S2.pdf]

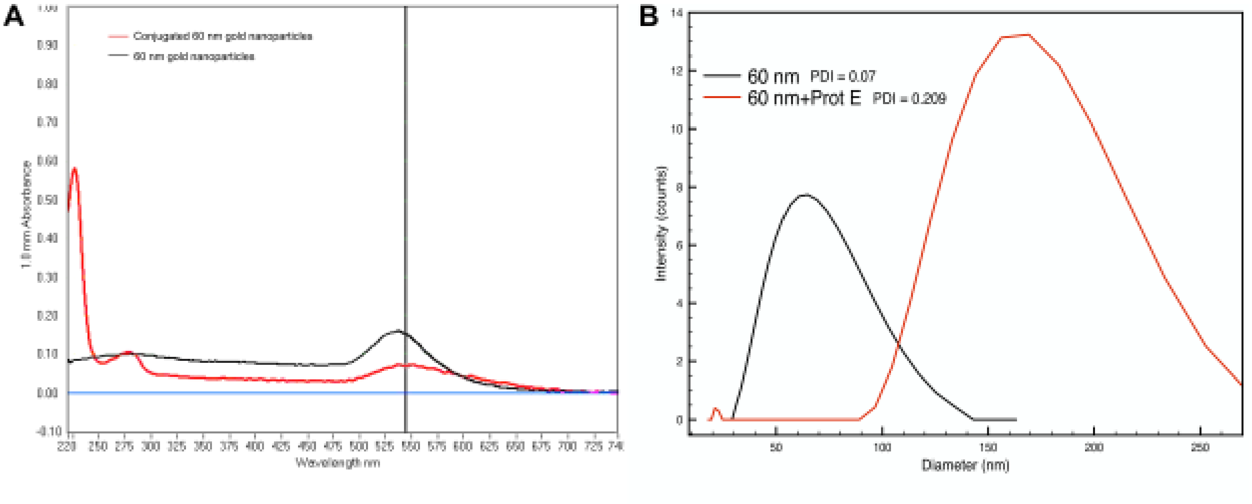

Figure S2. Characterization of the conjugated gold nanoparticles. a) Light extinction spectra and b) Hydrodynamic radii of 60 nm gold nanoparticles and Protein E conjugated gold nanoparticles. Polydispersity index (PDI) is indicated for each sample.
