# Supplementary material for: Feasibility Study of an Optical Caustic Plasmonic Light Scattering Sensor for Human Serum Anti-Dengue Protein E Antibody Detection
Source: Diagnostics (Basel). 2017 Aug 17;7(3):47. doi: 10.3390/diagnostics7030047 (PMC5617947; doi:10.3390/diagnostics7030047)
Supplement: Supplementary file 1 [file diagnostics-07-00047-s001.zip › diagnostics-212526-supplementary/Suppl Figures/Figure S3.pdf]

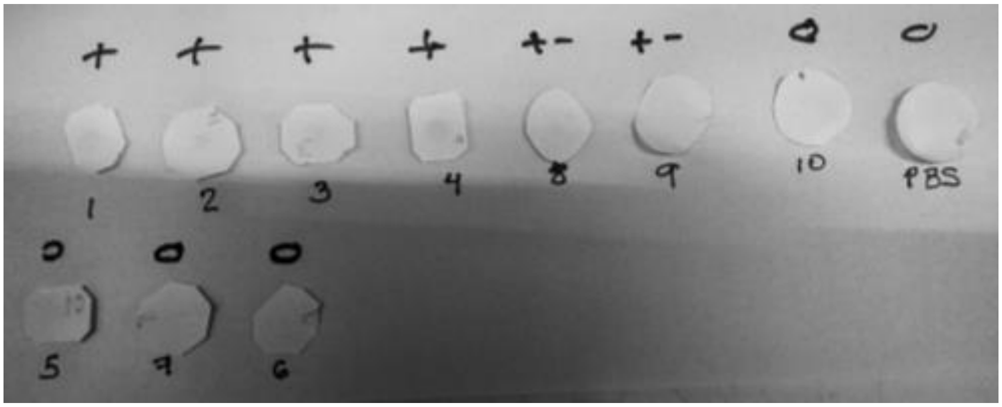

Figure S3. Image showing visual scoring of dot blots. Each blot was done in separate paper and reaction chambers in order to limit solution volume for gold conjugation and prevent cross contamination among the human serum samples and PBS negative control.
